# Genomic Sequencing from Sputum for Tuberculosis Disease Diagnosis, Lineage Determination, and Drug Susceptibility Prediction

**DOI:** 10.1128/jcm.01578-22

**Published:** 2023-02-23

**Authors:** Kayzad Nilgiriwala, Marie-Sylvianne Rabodoarivelo, Michael B. Hall, Grishma Patel, Ayan Mandal, Shefali Mishra, Fanantenana Randria Andrianomanana, Kate Dingle, Gillian Rodger, Sophie George, Derrick W. Crook, Sarah Hoosdally, Nerges Mistry, Niaina Rakotosamimanana, Zamin Iqbal, Simon Grandjean Lapierre, Timothy M. Walker

**Affiliations:** a Foundation for Medical Research, Mumbai, Maharashtra, India; b Mycobacteriology Unit, Institut Pasteur de Madagascar, Antananarivo, Madagascar; c European Molecular Biology Laboratory, European Bioinformatics Institute, Hinxton, Cambridgeshire, United Kingdom; d Department of Microbiology and Immunology, Peter Doherty Institute for Infection and Immunity, The University of Melbourne, Melbourne, Australia; e Nuffield Department of Clinical Medicine, John Radcliffe Hospital, Oxford University, Oxford, United Kingdom; f Immunopathology Axis, Centre de Recherche du Centre Hospitalier, Université de Montréal, Montréal, Québec, Canada; g Department of Microbiology, Infectious Diseases and Immunology, Université de Montréal, Montréal, Québec, Canada; h Oxford University, Clinical Research Unit, Ho Chi Minh City, Vietnam; Maine Medical Center Department of Medicine

**Keywords:** antimicrobial resistance, methods development, molecular epidemiology, tuberculosis, whole-genome sequencing

## Abstract

Universal access to drug susceptibility testing for newly diagnosed tuberculosis patients is recommended. Access to culture-based diagnostics remains limited, and targeted molecular assays are vulnerable to emerging resistance mutations. Improved protocols for direct-from-sputum Mycobacterium tuberculosis sequencing would accelerate access to comprehensive drug susceptibility testing and molecular typing. We assessed a thermo-protection buffer-based direct-from-sample M. tuberculosis whole-genome sequencing protocol. We prospectively analyzed 60 acid-fast bacilli smear-positive clinical sputum samples in India and Madagascar. A diversity of semiquantitative smear positivity-level samples were included. Sequencing was performed using Illumina and MinION (monoplex and multiplex) technologies. We measured the impact of bacterial inoculum and sequencing platforms on genomic read depth, drug susceptibility prediction performance, and typing accuracy. M. tuberculosis was identified by direct sputum sequencing in 45/51 samples using Illumina, 34/38 were identified using MinION-monoplex sequencing, and 20/24 were identified using MinION-multiplex sequencing. The fraction of M. tuberculosis reads from MinION sequencing was lower than from Illumina, but monoplexing grade 3+ samples on MinION produced higher read depth than Illumina (*P < *0.05) and MinION multiplexing (*P < *0.01). No significant differences in sensitivity and specificity of drug susceptibility predictions were seen across sequencing modalities or within each technology when stratified by smear grade. Illumina sequencing from sputum accurately identified 1/8 (rifampin) and 6/12 (isoniazid) resistant samples, compared to 2/3 (rifampin) and 3/6 (isoniazid) accurately identified with Nanopore monoplex. Lineage agreement levels between direct and culture-based sequencing were 85% (MinION-monoplex), 88% (Illumina), and 100% (MinION-multiplex). M. tuberculosis direct-from-sample whole-genome sequencing remains challenging. Improved and affordable sample treatment protocols are needed prior to clinical deployment.

## INTRODUCTION

Before the coronavirus disease 2019 pandemic, tuberculosis (TB) was responsible for more deaths than any other infectious disease. In addition, notified cases of TB dropped by 18% between 2019 and 2020, coinciding with the pandemic's onset (1). This decline was not because the caseload was reduced but because TB diagnosis capacity was severely disrupted. Control of this much older pandemic has thereby been set back a decade (2). Although the impact on health systems has been an acute issue, diagnostic capacity within those systems was already underserving population needs in many low- and middle-income settings before the pandemic.

Gold standard culture-based diagnostics remain out of reach for many patients with TB, as these diagnostics are dependent on centralized laboratory infrastructures, technically demanding, and expensive. Molecular platforms such as Xpert MTB/RIF Ultra have delivered the capacity to confirm the presence of Mycobacterium tuberculosis and to predict most resistance to rifampin from primary clinical samples in settings that had previously relied on smear microscopy only (3). Nevertheless, as such targeted molecular assays are vulnerable to off-target emerging mutations and provide limited information on susceptibility to other drugs, treatment for many patients remains semiempirical, with an increased risk of treatment failure and amplification of resistance to more drugs (4). Whole-genome sequencing (WGS) has been heralded as a potential solution for the implementation of personalized therapy, but there remains a need for a culture amplification step prior to sequencing, and solutions have so far proved stubbornly out of reach (5, 6).

Although targeted next-generation sequencing (tNGS) can identify species and lineage, predict drug susceptibility, and inform on spoligotype, it is inherently restricted in its resolution in comparative genomics for outbreak investigations compared to WGS. WGS, therefore, remains the ultimate goal (7). Here, we explored how close we could get to obtaining useful diagnostic information from sequencing of primary clinical samples in two high-burden settings: in Madagascar, a low-income country, and in India, a middle-income country. We present data on samples with a range of bacillary loads, sequenced on both laboratory-confined (Illumina) and portable (Oxford Nanopore Technologies [ONT]) sequencing platforms to assess how close we are to implementing culture-free sequencing protocols into the clinical space, where they are most needed and could critically reduce TB diagnostics turnaround times.

## MATERIALS AND METHODS

### Study design and sampling.

The study was designed to assess direct-from-sample whole-genome sequencing on Illumina (NextSeq 500 or HiSeq 2500) and ONT MinION Mk 1B sequencing platforms, according to smear microscopy grade, with and without multiplexing on MinION flow cells. The study was conducted at the Foundation for Medical Research (Mumbai, India) and at the Antituberculosis Dispensary Center (DAT, in Antananarivo, Madagascar). The study protocol determined that the first 10 patient samples should be collected from each center for each WHO smear microscopy grade (1+, 2+, 3+). Each sample also needed to be positive for M. tuberculosis on the Xpert MTB/RIF Ultra system (Cepheid, USA). Six smear-negative, Xpert MTB/RIF Ultra-negative samples were collected from each center to use as controls. In India, these samples were from healthy volunteers, and in Madagascar, they were from patients with other pulmonary pathologies. All samples were divided by pipette into two equal-volume samples. One aliquot was set up for culture and one aliquot was used directly for DNA extraction. All samples were processed on the collection day or the following day after overnight storage at 4°C. DNA extracted directly from clinical samples was divided in two for WGS on each platform. For WGS on MinION, five samples were multiplexed and five monoplexed at each center for each smear grade, along with negative controls. In each case, two multiplexed samples were also monoplexed for cross-validation (Table 1). Outcome measures included sequencing mean read depth, species and lineage identification, and accuracy of genotypic drug susceptibility results compared to culture-based WGS.

**TABLE 1:** Study samples^*a*^

Technology	Sample type	Smear microscopy category	Madagascar result, by smear grade				Mumbai result, by smear grade				Total smear positive (%)
			−	1+	2+	3+	−	1+	2+	3+	
All		Total no. of samples collected	6	10	10	10	6	10	10	10	60
Xpert	Sputum	Tested by Xpert Ultra	6	10	9	10	5	10	10	10	59
		Xpert assay failed	0	1	0	0	0	0	0	0	1
		M. tuberculosis detected by Xpert	0	9	9	10	0	10	10	10	58
Illumina	Culture	Samples set up for culture	6	10	10	10	6	10	10	10	60
		Culture positive and WGS result	0	6	7	10	0	5	8	8	44
		M. tuberculosis detected from WGS	0	6	7	10	0	5	8	8	44 (100)
		Lineage determined from WGS	0	6	7	10	0	5	8	8	44 (100)
Illumina	Sputum	Samples sequenced	6	10	10	9	6	5	8	9	51
		M. tuberculosis detected from WGS	1	7	9	7	6	5	8	9	45 (88)
		Lineage called	0	4	6	5	0	2	6	4	27 (53)
		Lineage from sputum was same as lineage from culture	0	3/3	4/6	5/5	0	2/2	3/6	3/4	20/26 (77)
Nanopore Monoplex	Sputum	Samples sequenced	3	7	7	7	3	5	6	6	38
		M. tuberculosis detected from WGS	0	5	6	7	0	4	6	6	34 (89)
		Lineage called	0	2	1	5	0	3	5	5	21 (55)
		Lineage from sputum was same as lineage from culture	0	2/2	1/1	5/5	0	2/3	5/5	2/4	17/20 (85)
Nanopore Multiplex	Sputum	Samples sequenced	3	5	5	5	3	0	4	5	24
		M. tuberculosis detected from WGS	0	4	4	3	0	0	4	5	20 (83)
		Lineage called	0	1	0	0	0	0	2	2	5 (21)
		Lineage from sputum was same as lineage from culture	0	1/1	0	0	0	0	2/2	2/2	5/5 (100)

a Data include the number of samples, samples for which M. tuberculosis was detected and for which lineage identification was achieved for each combination of sequencing technology and sequencing approach (culture versus sputum).

### Sample processing.

Clinical samples containing minimally 1 mL of sputum were liquefied using an equal volume of thermo-protection buffer containing dithiothreitol (4 M KCl, 0.05 M HEPES buffer, pH 7.5, 0.1% dithiothreitol [DTT]) (8). One-milliliter aliquots were heat inactivated in 2-mL screw-cap tubes at 99°C for 30 min. Samples were centrifuged (6,000 × *g* for 3 min) and resuspended in 100 μL phosphate-buffered saline. DNA was extracted as previously described and quantified by fluorometry (Qubit dsDNA HS assay kit, Thermo Fisher Scientific, USA) (8).

Nanopore sequencing was performed locally in each center. DNA libraries were prepared using a ligation kit (SQK-LSK 109) and sequenced on MinION devices using R9.4 flow cells. Sequencing was conducted for 48 to 72h, and data were acquired with MinKNOW (v19.0). In addition, the native barcoding expansion kit EXP-NBD104 (Oxford Nanopore Technologies, United Kingdom) was used for multiplexing on single-flow cells. DNA concentrations were normalized to avoid overrepresentation of samples in the library preparation steps for Illumina and Nanopore multiplex sequencing.

### Sequencing data processing.

Guppy (v5.0.16) was used to demultiplex and base-call MinION data, keeping only default “pass” reads with a mean quality score of at least 7. Illumina data were preprocessed with fastp (v0.23.2) to remove adapter sequences, trim low-quality bases from read ends, and remove reads shorter than 30 bp (9). Sequencing reads were decontaminated and mapped to a database of common sputum contaminants and to the M. tuberculosis reference genome (H37Rv [NC_000962.3]), keeping only those reads with a mapping to H37Rv (10, 11). Mapping was performed with bwa mem (v0.7.17; parameters M) for Illumina data and minimap2 (v2.23; parameters ax, map-ont) for Nanopore (12, 13). Decontaminated sequence files for each isolate were randomly subsampled with Rasusa (v0.6.0) to a maximum (mean) read depth of 100 (Illumina) and 150 (MinION) (14). The composition of each isolate’s sequencing data was determined by matching each read’s best mapping in the contamination alignment file to a classification category of M. tuberculosis, nontuberculous mycobacteria (NTM), other bacteria, virus, human, or unmapped. Mykrobe (v.0.10.0) was used to assign lineage and predict drug susceptibility using parameters described in Hall et al. (10, 11, 15). The complete list of gene targets and the resistance conferring mutation catalog used by Mykrobe was previously reported and is available online and in Tables S1 and S2 in the supplemental material (10, 16).

### Drug susceptibility testing.

Phenotypic susceptibility testing was performed using a UKMYC6 broth microdilution plate (Mumbai) and Lowenstein-Jensen (LJ) proportion method (Antananarivo) (17, 18). This was done as part of country- and patient-specific routine TB care. Results were not available for all genotypically tested drugs, and drugs for which phenotypic results were available varied between samples. Phenotypic drug susceptibility testing (DST) results were hence not used in the analysis but are provided in Table S2. Culture-based WGS on Illumina results was used for reference standards, as that was what we were trying to replicate by performing WGS directly from clinical samples.

### Statistics.

A Wilcoxon rank-sum test was used to compare DNA concentration, read depth, and accuracy of predicted outcome by smear grade and sequencing platform. Linear regression was used to assess the relationship between DNA concentration and mean read depth of the genome. Fischer’s exact test was used to compare the pooled sensitivity and specificity for DST predictions to drugs of interest across each sequencing modality.

### Data availability.

The decontaminated read (FASTQ) files for each isolate have been deposited in the European Nucleotide Archive under project accession number PRJEB56100. A data sheet mapping accession numbers to study information can additionally be found at https://doi.org/10.6084/m9.figshare.21193588. The code used to perform all analyses in this study is available at https://github.com/iqbal-lab-org/tb-sputum-project.

## RESULTS

Between September 2019 and January 2020, each of the two study centers collected 10 sputum samples for each smear microscopy grade (1+, 2+, 3+) along with six negative controls, for a grand total of 72 included samples. All samples were set up for culture. Fifty-eight of 60 microscopy-positive samples were confirmed as M. tuberculosis by Xpert MTB/RIF Ultra. Testing was not performed for one sample and failed for another. For both those samples, culture and whole-genome sequencing (from sputum and culture) confirmed samples as TB positive, and they were hence included in the analysis. Eleven of 12 control samples were negative for M. tuberculosis by Xpert MTB/RIF Ultra and 1 was not tested, but all negative controls were negative in culture.

All 30 smear-positive samples in Madagascar were positive for TB by culture, but Illumina WGS-based genotypic DST results were only available for 23 samples because of DNA extraction quality control issues. In India, 24 were positive in MGIT, and Illumina WGS from culture was available for 21. For all 44 samples sequenced from culture, M. tuberculosis and lineage were identified from the WGS data. Aliquots from the corresponding sputum samples, those that did not grow in MGIT, and negative controls were used to extract DNA directly for WGS on an Illumina platform without a culture step. Sufficient DNA was obtained for WGS from 51/60 smear-positive samples. Among these, M. tuberculosis was identified from 45/51 (88%) samples, with no species reported for the remaining six, and a lineage was identified from 27/51 (53%). Where a lineage or sublineage was called from both culture- and sputum-based WGS, 20/26 (77%) agreed at the lineage level (Table 1). However, in 6/6 and 1/6 negative controls sequenced from sputum on Illumina in India and Madagascar, respectively, M. tuberculosis reads were identified, albeit a very small amount (<0.002% reads).

A subset of these primary sputum samples was further sequenced on MinION platforms. Thirty-eight smear-positive samples were sequenced individually (one per flow cell, i.e., monoplex), and 24 were multiplexed. M. tuberculosis was identified from 34/38 (89%) monoplexed samples and from 20/24 (83%) multiplexed samples, and a lineage was called for 21/38 (55%) and 5/24 (21%) samples, respectively. Where a lineage or sublineage was called from both culture- and sputum-based WGS on MinION monoplex and multiplex, 17/20 (85%) and 5/5 (100%), respectively, agreed at the lineage level. All negative controls sequenced on MinION were negative for M. tuberculosis.

**FIG 1:**
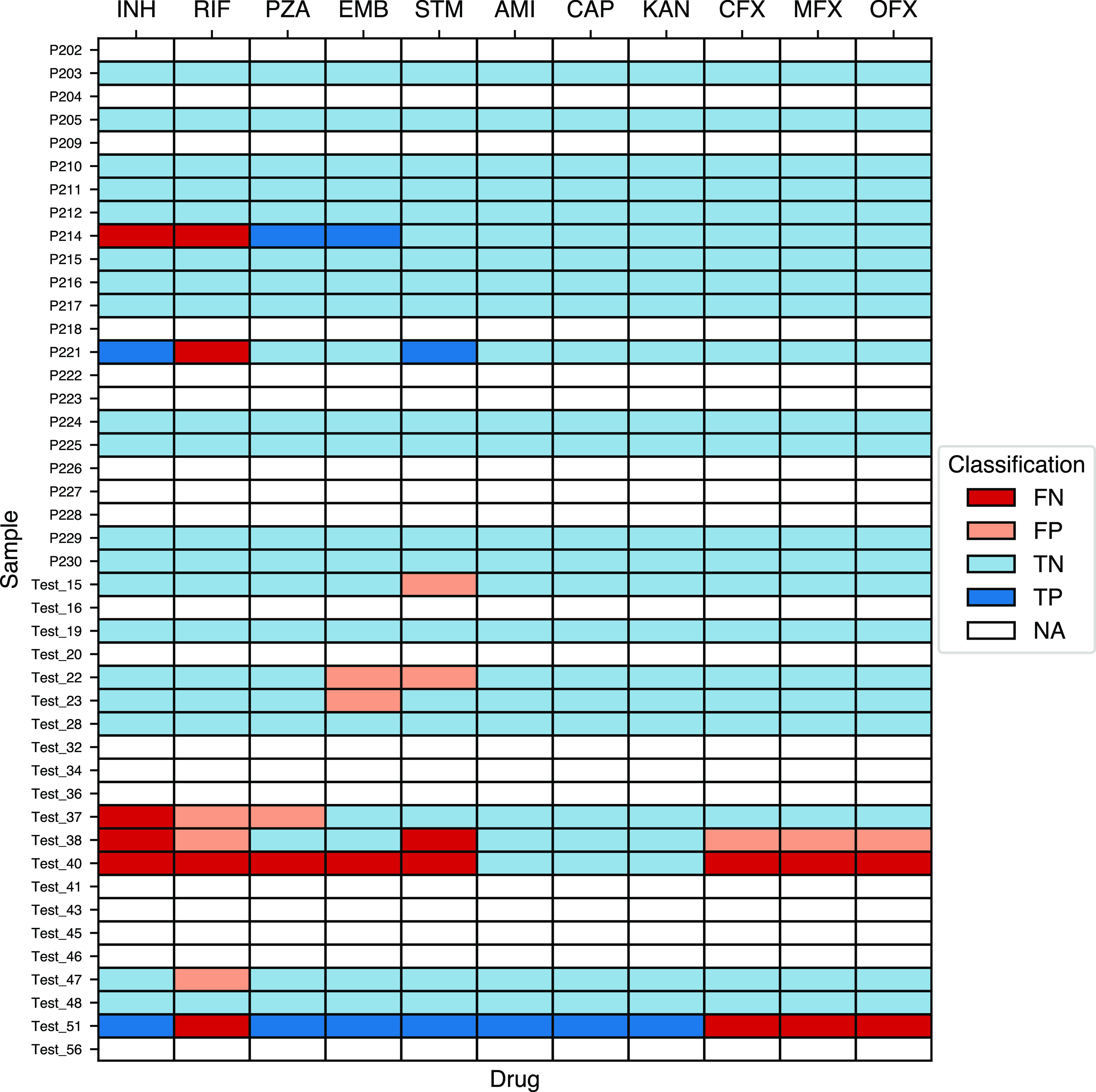
Drug susceptibility testing accuracy of Illumina-based direct-from-sputum sequencing using culture-based WGS on Illumina results as the reference standard. Note that only those samples with culture-based Illumina sequencing are shown. FN, false negative; FP, false positive; TP, true positive; TN, true negative; NA, data not available.

**FIG 2:**
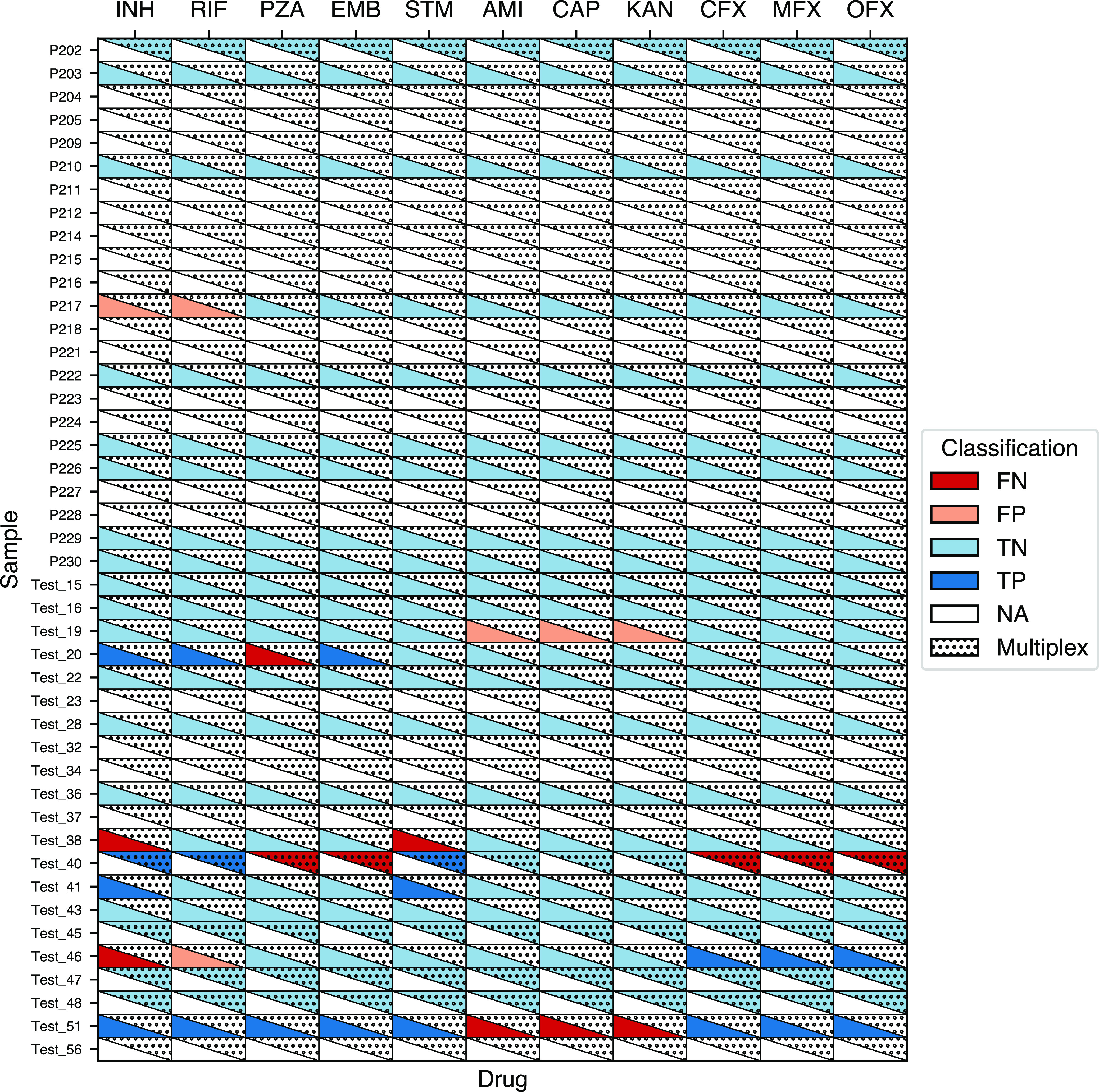
Drug susceptibility testing accuracy of nanopore MinION-based direct-from-sputum sequencing using culture-based WGS on Illumina results as the reference standard. For each sample, predictions from monoplexing and multiplexing (triangles with dots) are presented when available. Note that only those samples with culture-based Illumina sequencing are shown. FN, false negative; FP, false positive; TP, true positive; TN, true negative; NA, data not available.

The reference for drug susceptibility predictions was culture-based WGS on Illumina, as that was what we were trying to replicate by performing WGS directly from clinical samples. Unfortunately, our collections included few resistant isolates, making sensitivity hard to assess. Nevertheless, resistance was correctly detected from clinical samples sequenced directly from sputum on Illumina for 6/12 (50%) isoniazid-resistant samples and 4/7 (57%) streptomycin-resistant samples. Only 1/8 (13%) rifampin-resistant samples were detected, and 0/4 moxifloxacin-resistant samples was detected. All isolates found to be rifampin-resistant by Illumina sequencing from culture were also resistant on Xpert Ultra testing except one for which no Xpert Ultra result was available. The number of resistant samples sequenced on MinION, either monoplex or multiplex, was lower. However, specificity was generally high, over 90% for all estimates other than for rifampin on Illumina (81%) (Table 2; Fig. 1 and 2). To assess the impact of smear microscopy grade and WGS method (Illumina, MinION monoplex or multiplex) on DST prediction from sputum, we pooled the predictions across drugs. No significant differences in overall sensitivity or specificity were seen across these sequencing modalities or within each modality when stratified by smear grade (Table 2).

**TABLE 2:** Drug susceptibility prediction agreement between standard Illumina sequencing from culture and each combination of sputum-based sequencing technology^*a*^

Technology and drug	S based on Illumina culture and comparison DST result, by smear grade								R based on Illumina culture and comparison DST result, by smear grade								Sensitivity (95% CI)	Specificity (95% CI)	*P* value^*b*^		
	S				R				S				R								
	1+	2+	3+	Total	1+	2+	3+	Total	1+	2+	3+	Total	1+	2+	3+	Total			Comparison	SENS	SPEC
Illumina for sputum																					
Isoniazid	5	10	10	25	0	0	2	2	2	3	1	6	2	2	2	6	50 (21–79)	93 (76–99)			
Rifampin	5	11	9	25	0	2	4	6	3	2	2	7	1	0	0	1	13 (0–53)	81 (63–93)			
Ethambutol	4	12	14	30	1	1	0	2	3	1	0	4	1	1	1	3	43 (10–82)	94 (79–99)			
Pyrazinamide	6	13	13	32	0	0	1	1	3	1	0	4	0	1	1	2	33 (4–87)	97 (84–100)			
Moxifloxacin	8	12	14	34	0	1	0	1	1	2	1	4	0	0	0	0	0 (0–60)	97 (85–100)			
Streptomycin	6	11	13	30	1	1	0	2	1	2	0	3	1	1	2	4	57 (18–90)	94 (79–99)			
Amikacin	8	15	14	37	1	0	0	1	0	0	0	0	0	0	1	1		97 (86–100)			
Total	42	84	87	213	3	5	7	15	13	11	4	28	5	5	7	17					
																			1+ vs 3+	0.31	1
																			2+ vs 3+	0.49	1
																			1+ vs 2+	1	1
Nanopore monoplex																					
Isoniazid	5	6	10	21	0	1	1	2	1	2	0	3	1	1	1	3	50 (12–88)	91 (72–99)			
Rifampin	5	8	11	24	0	2	0	2	1	0	0	1	1	0	1	2	67 (9−99)	92 (75–99)			
Ethambutol	5	10	11	26	0	0	0	0	1	0	0	1	1	0	1	2	67 (9–99)	100 (87–100)			
Pyrazinamide	5	9	11	25	0	1	0	1	2	0	0	2	0	0	1	1	33 (1–90)	96 (80–100)			
Moxifloxacin	7	9	11	27	0	0	0	0	0	0	0	0	0	1	1	2	100 (16–100)	100 (87–100)			
Streptomycin	6	8	11	25	0	0	0	0	1	1	0	2	0	1	1	2	50 (7–93)	100 (86–100)			
Amikacin	6	10	11	27	1	0	0	1	0	0	1	1	0	0	0	0		96 (82–100)			
Total	39	60	76	175	1	4	1	6	6	3	1	10	3	3	6	12					
																			1+ vs 3+	0.41	1
																			2+ vs 3+	0.67	0.9
																			1+ vs 2+	1	1
																			All vs all (Illumina from sputum)	0.45	0.83
Nanopore multiplex																					
Isoniazid	3	5	6	14	0	1	0	1	0	1	2	3	0	1	0	1	25 (1–81)	93 (68–100)			
Rifampin	3	6	7	16	0	0	0	0	0	1	1	2	0	1	0	1	33 (1–91)	100			
Ethambutol	3	6	8	17	0	0	0	0	0	2	0	2	0	0	0	0	0 (0–71)	100			
Pyrazinamide	3	6	8	17	0	0	0	0	0	2	0	2	0	0	0	0	0 (0–84)	100			
Moxifloxacin	3	7	8	18	0	0	0	0	0	1	0	1	0	0	0	0	0 (0–98)	100			
Streptomycin	3	7	7	17	0	0	0	0	0	0	1	1	0	1	0	1	100 (3–100)	100			
Amikacin	3	8	8	19	0	0	0	0	0	0	0	0	0	0	0	0		100			
Total	21	45	52	118	0	1	0	1	0	7	4	11	0	3	0	3					
																			1+ vs 3+		1
																			2+ vs 3+	0.51	1
																			1+ vs 2+		1
																			All vs all (Illumina from sputum)	0.76	0.75

a Genotypic drug susceptibility testing predictions from direct sputum sequencing were only made for samples where M. tuberculosis was identified. R, resistant; S, susceptible; CI, confidence interval.

b Determined with Fisher’s exact test and based on numbers pooled across all drugs within a given sequencing modality. Comparisons were between the indicated smear grades and the corresponding sensitivity (SENS) and specificity (SPEC).

**FIG 3:**
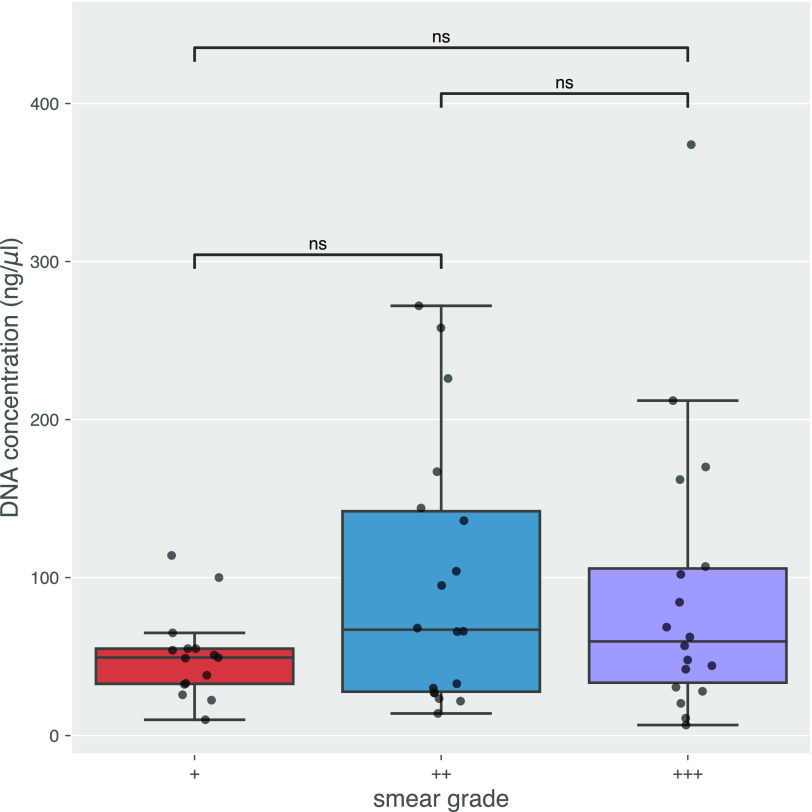
DNA concentration (*y* axis) for each smear grade (*x* axis). Each point represents a single sample. Annotated *P* values were calculated with a Wilcoxon rank-sum test. ns, not significant.

**FIG 4:**
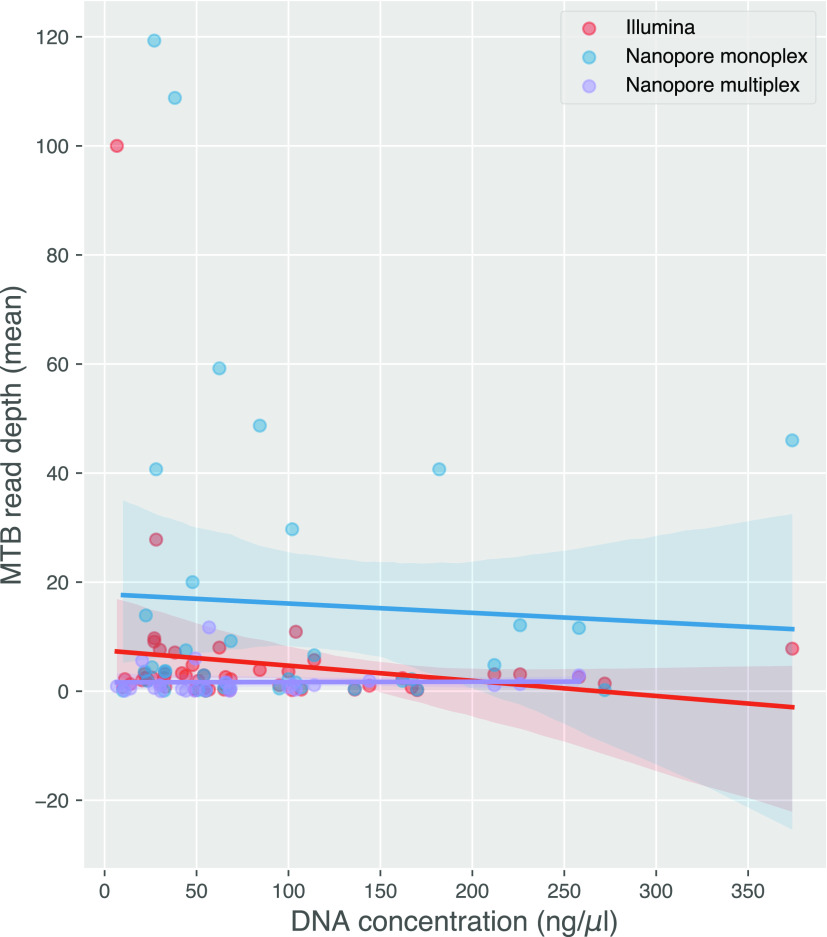
Relationship between mean read depth on the M. tuberculosis genome (*y* axis) and the concentration of DNA extracted from the isolate (*x* axis) for all three sequencing approaches. The shaded areas represent the 95% confidence intervals. Each point represents a sample-sequencing strategy pair, and so some samples appear multiple times.

**FIG 5:**
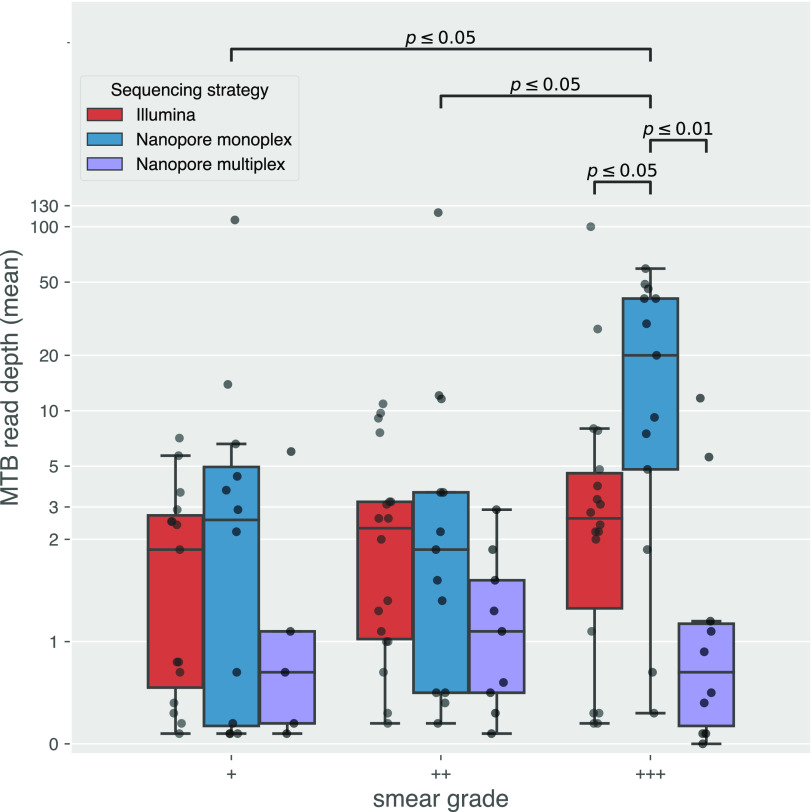
Mean read depth on the M. tuberculosis genome (*y* axis), stratified by smear grade (*x* axis) and sequencing strategy (colors). Each point represents a single sample. Annotated *P* values were calculated with a Wilcoxon rank-sum test.

**FIG 6:**
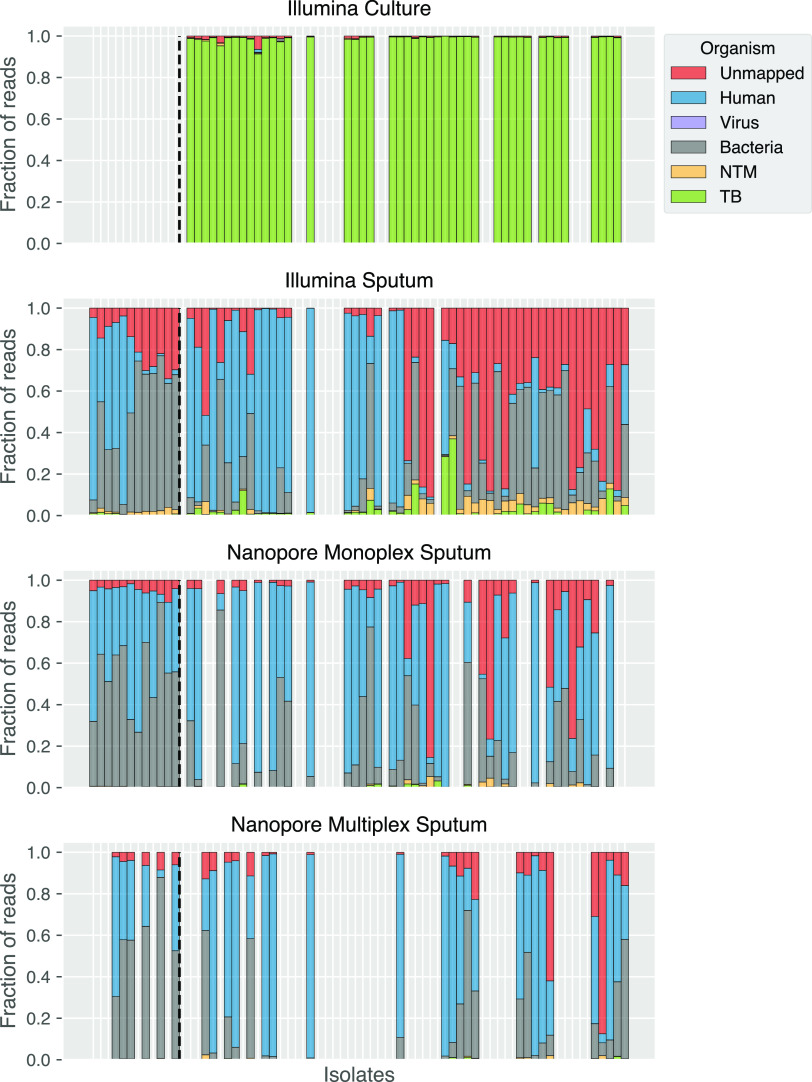
Fraction of reads (*y* axis) from sequencing strategies that aligned to given organisms (colors). Each bar represents a single sample. Bars are vertically aligned such that the same isolate has the same place on the *x* axis across the sequencing strategies. Where a bar is missing, that isolate was not sequenced with that strategy. Bars to the left of the vertical black dashed line are negative controls. Note that most sputum isolates have TB reads, but the fraction is so low it is sometimes not possible to see it. NTM, nontuberculous mycobateria; TB, Mycobacterium tuberculosis; NA, sequencing not available.

Next, we sought to understand the relationship between smear grade, DNA concentration after extraction, read depth, and accuracy of predicted outcomes (species, lineage, and DST). There was no significant increase in DNA concentration among samples with different smear grades (Fig. 3). M. tuberculosis read depth did not increase with higher DNA concentrations for Nanopore monoplex sequencing where DNA inputs were not normalized. For Nanopore multiplex and Illumina where DNA inputs were normalized at the library preparation step, prenormalization DNA concentration did not predict M. tuberculosis read depth either (Fig. 4). Only the smear grade 3+ monoplexed samples sequenced on MinION showed a significant increase in M. tuberculosis read depth (*P < *0.05 to grades 1+ and 2+) (Fig. 5). Looking across sequencing modalities by smear grade, we saw that monoplexing on MinION for grade 3+ sputum samples produced the highest read depth (*P < *0.05 compared to Illumina and *P < *0.01 compared to the MinION multiplex) (Fig. 5). Interestingly, the fraction of M. tuberculosis reads from MinION sequencing was lower than that from Illumina (Fig. 6), even though it had greater read depth (monoplexed).

**FIG 7:**
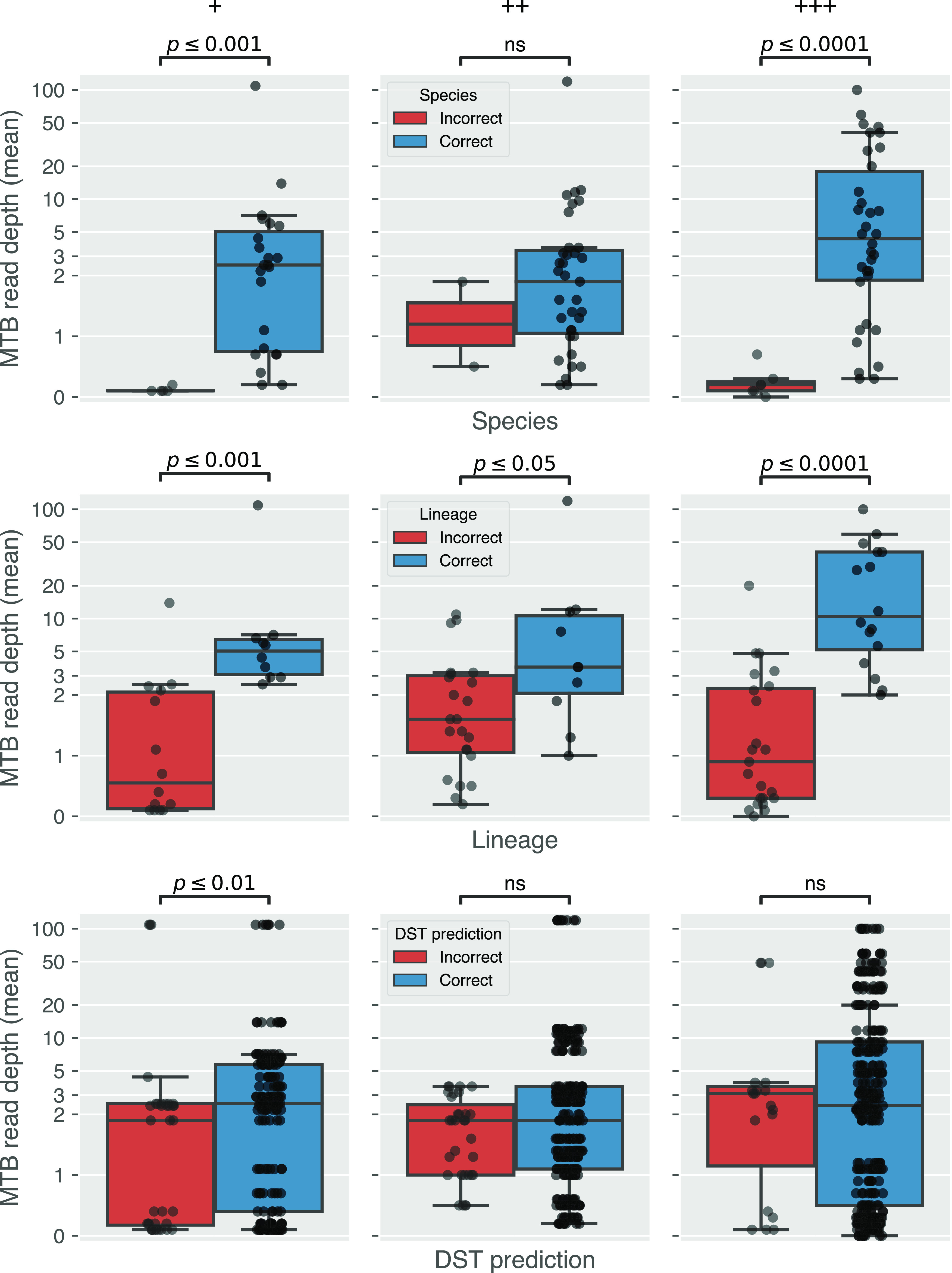
Impact of mean read depth (*x* axis) on predicting species (top panel), lineage (middle panel), and DST result (bottom panel). Samples are stratified by whether the prediction was correct (blue) or not (red). Note that for DST each point is a sample-drug pair and hence why it has more points than species and lineage. Annotated *P* values were calculated with a Wilcoxon rank-sum test.

Despite the limited evidence for increased M. tuberculosis read depth with DNA concentration or smear grade, there was clear evidence that increased read depth led to improved predictions of both species and lineage (Fig. 7). For DST, this was only apparent for smear grade 1+ sputum samples (*P < *0.01) and not for grades 2+ and 3+ samples (Fig. 7).

## DISCUSSION

This study aimed to derive clinically useful information from WGS performed on primary clinical samples in two high-TB-burden settings. We compared results from Illumina and ONT MinION platforms, using monoplex and multiplex approaches on MinION, across various sputum smear microscopy grades. Using Illumina WGS data from the cultured isolates as a reference, we assessed how well we could replicate culture-based WGS predictions for M. tuberculosis species, lineage, and drug susceptibility from sputum samples.

Given how challenging direct-from-sample WGS still is for M. tuberculosis, it is unsurprising that the results are not yet good enough for clinical deployment. Although species could be correctly called for 83 to 89% of samples when sequenced directly from sputum, a lineage could only be called for 21 to 55%, although it was mostly called correctly in those samples. Drug susceptibility predictions were also challenging, with sensitivity often around 50% or lower and no drugs performing consistently well. Specificity was generally high—only Illumina sputum rifampin predictions (81%) had a value lower than 90%—but that was likely a function of the predominance of susceptible strains in the collection, which was a study limitation. Future improved sample processing protocols should be evaluated on sputum collected from studies purposively designed to include an increased number of drug-resistant isolates. In our study, no specific steps were taken to ensure that aliquots included equal mycobacterial inoculums. Results across platforms might hence have been impacted by inadvertent unequal splitting of samples, which was also a limitation.

We aimed to assess an inexpensive approach which could be deployed in low- and middle-income settings. Brown et al. previously used a biotinylated RNA baits approach and recovered over 98% of the M. tuberculosis genome from 20 of 24 (83%) of smear-positive, culture-positive sputa included in their study (19). This approach outperformed ours but is significantly more expensive and hence is not foreseen as being widely implemented in the context of high-TB-burden countries.

It is a reasonable assumption that the per-sample cost of WGS will likely be affected by whether the samples are monoplexed or multiplexed on a MinION. We unsurprisingly obtained greater read depth by monoplexing than multiplexing. However, we did not find that read depth from multiplexed MinION samples differed from those sequenced on Illumina, suggesting that with improved approaches to enriching mycobacterial DNA there may be a future for multiplex WGS on MinION. In our hands, a long-read sequencing approach added no value over or above short-read sequencing. Indeed, it has been well established that M. tuberculosis genotypic DST and molecular typing can be accurately performed using short reads. Long-read sequencing may play a more important role in the future if relevant targets are discovered that cannot be reliably mapped using short reads.

The number of negative controls from which M. tuberculosis reads were identified after Illumina sequencing was concerning, especially if culture-free sequencing is to be used simultaneously for TB diagnosis and DST. In our study, flow cells were only used once for a full sequencing experiment, so cross-contamination was not due to flow cell washing and reuse, although this should be monitored if such an approach is adopted in the future. It is clear that the risk of cross-contamination is substantial and will only grow as higher concentrations of DNA are used. This emphasizes the need to follow standard good practices for molecular laboratory DNA contamination prevention. Individually processing samples and preparing libraries with barcoding may help control cross-contamination. Potential approaches may also involve bioinformatics solutions, such as establishing more specific read mapping thresholds.

Interestingly, there was minimal evidence of a relationship between smear grade, DNA concentration when extracting directly from sputum samples, and mean read depth across the genome. This held true for Nanopore monoplex sequencing, where input DNA concentrations were not normalized prior to sequencing. Read depth was nevertheless a decisive factor in determining the correct species, lineage, and to some extent drug susceptibility predictions. The implication is that it might be challenging to use the smear grade or DNA concentration after extraction to predict whether valuable results will likely emerge from sequencing a given specimen.

One of the main challenges with performing WGS directly from clinical samples is that the amount of extractable mycobacterial DNA is tiny compared with that from human and nonmycobacterial microorganisms. This was reflected in the meager fraction of reads mapping to M. tuberculosis. Culture amplifies this fraction enormously, with the cost of slowing the diagnostic process. Performing WGS directly from clinical samples remains desirable even though targeted next-generation sequencing can identify species and lineage and predict DST by amplifying multiple molecular targets (20). Although it can also be used for spoligotyping, the resolution of spoligotyping for comparative genomics is intrinsically limited compared to that obtained by WGS, which enables transmission studies and may accelerate infection control interventions (21, 22). Also, obtaining information on genomic variants which are outside the targets of PCR-based or tNGS assays could inform on potential new candidate resistance mutations and prospectively enrich geno-to-pheno resistance databases, especially for newly released drugs. Obtaining all that information from a single sputum-based assay in a timely, reliable, and cost-effective manner would hence represent a significant advance.

We have assessed how well WGS direct from sputum performs in our hands when applied in high-TB-burden, low- and middle-income settings. We focused on predicting species, lineage, and DST rather than performing comparative genomics, and we showed that even for these relatively more straightforward tasks, there remains much room for improvement. Whole-genome amplification approaches may yield better results than reported here and should sensibly be pursued in the future.
