# Supplementary material for: Genomic Sequencing from Sputum for Tuberculosis Disease Diagnosis, Lineage Determination, and Drug Susceptibility Prediction
Source: J Clin Microbiol. 2023 Feb 23;61(3):e01578-22. doi: 10.1128/jcm.01578-22 (PMC10035339; doi:10.1128/jcm.01578-22)
Supplement: Supplemental file 1 — Supplemental material. Download jcm.01578-22-s0001.pdf, PDF file, 0.07 MB [file jcm.01578-22-s0001.pdf]

Supplementary material 1

Gene	Drug Target
ahpC	Isoniazid
eis	Kanamycin
embA	Ethambutol
embB	Ethambutol
fabG1	Isoniazid
gid	Streptomycin
gyrA	Ofloxacin
gyrA	Moxifloxacin
gyrA	Ciprofloxacin
inhA	Isoniazid
katG	Isoniazid
pncA	Pyrazinamide
rpoB	Rifampicin
rpsL	Streptomycin
rrs	Capreomycin
rrs	Kanamycin
rrs	Amikacin
rrs	Streptomycin

Supplementary material 1 – Complete list of gene targets and associated drugs which are included in the resistance conferring mutation catalog used by Mykrobe.
